# A Latitudinal Diversity Gradient in Terrestrial Bacteria of the Genus *Streptomyces*

**DOI:** 10.1128/mBio.02200-15

**Published:** 2016-04-05

**Authors:** Cheryl P. Andam, James R. Doroghazi, Ashley N. Campbell, Peter J. Kelly, Mallory J. Choudoir, Daniel H. Buckley

**Affiliations:** School of Integrative Plant Science, Cornell University, Ithaca, New York, USA

## Abstract

We show that *Streptomyces* biogeography in soils across North America is influenced by the regional diversification of microorganisms due to dispersal limitation and genetic drift. *Streptomyces* spp. form desiccation-resistant spores, which can be dispersed on the wind, allowing for a strong test of whether dispersal limitation governs patterns of terrestrial microbial diversity. We employed an approach that has high sensitivity for determining the effects of genetic drift. Specifically, we examined the genetic diversity and phylogeography of physiologically similar *Streptomyces* strains isolated from geographically distributed yet ecologically similar habitats. We found that *Streptomyces* beta diversity scales with geographic distance and both beta diversity and phylogenetic diversity manifest in a latitudinal diversity gradient. This pattern of *Streptomyces* biogeography resembles patterns seen for diverse species of plants and animals, and we therefore evaluated these data in the context of ecological and evolutionary hypotheses proposed to explain latitudinal diversity gradients. The data are consistent with the hypothesis that niche conservatism limits dispersal, and historical patterns of glaciation have limited the time for speciation in higher-latitude sites. Most notably, higher-latitude sites have lower phylogenetic diversity, higher phylogenetic clustering, and evidence of range expansion from lower latitudes. In addition, patterns of beta diversity partition with respect to the glacial history of sites. Hence, the data support the hypothesis that extant patterns of *Streptomyces* biogeography have been driven by historical patterns of glaciation and are the result of demographic range expansion, dispersal limitation, and regional diversification due to drift.

## IMPORTANCE

Biogeographic patterns provide insight into the evolutionary and ecological processes that govern biodiversity. However, the evolutionary and ecological processes that govern terrestrial microbial diversity remain poorly characterized. We evaluated the biogeography of the genus *Streptomyces* to show that the diversity of terrestrial bacteria is governed by many of the same processes that govern the diversity of many plant and animal species. While bacteria of the genus *Streptomyces* are a preeminent source of antibiotics, their evolutionary history, biogeography, and biodiversity remain poorly characterized. The observations we describe provide insight into the drivers of *Streptomyces* biodiversity and the processes that underlie microbial diversification in terrestrial habitats.

## INTRODUCTION

Patterns of microbial biogeography remain ill described because tools for measuring microbial diversity have emerged only recently and are evolving rapidly. It is still unclear to what degree patterns of microbial diversity can be explained by the same ecological and evolutionary forces that govern the diversity of plants and animals. Evolutionary ecology seeks to explain the forces that govern evolutionary diversification in an ecological context. If the same forces govern the diversification of both macro- and microorganisms, then it will be possible to bring the full theoretical framework which underpins evolutionary ecology to bear in evaluating the causes and consequences of microbial diversity.

Microorganisms, due to their small size, large populations, ability to survive dormancy, and potential for wind dissemination (1), may support rates of dispersal that far exceed rates of diversification (2–4). If dispersal rates greatly exceed diversification rates, then microbial diversity is controlled by selection acting on global scales (5). There is now abundant evidence that environmental gradients explain patterns of microbial beta diversity on local and regional scales (for reviews, see references 5, 6, and 7). It remains unclear, however, to what degree these biogeographic patterns are the result of recruitment from globally distributed species pools or from dispersal limitation and evolutionary diversification acting on regional scales.

Microorganisms lack morphological features to enable identification, and definitions for microbial species remain controversial (8). Patterns of microbial biogeography are often inferred from the analysis of small-subunit (SSU) rRNA genes, with operational taxonomic units (OTU) defined using a 3% nucleotide identity cutoff (9). The SSU rRNA gene, however, has an exceptionally low substitution rate, and it is estimated that approximately 50 million years are required to achieve 1% divergence in SSU rRNA sequences (10). As a point of comparison, the family *Leguminosae* first appeared approximately 60 million years ago (11), and hence all legumes would represent a single OTU if plant diversity was defined in the manner of microbial diversity. Biogeographic patterns detected through the analysis of SSU rRNA genes likely result from physiological traits that map deeply in the tree of life. Indeed, some of the best-documented patterns of microbial biogeography have been made at the phylum level. For example, *Betaproteobacteria* are common in freshwater environments but uncommon in saltwater; *Actinobacteria* and *Firmicutes* are ubiquitous in soils but rare in oceans, and *Actinobacteria* decrease in abundance in acidic soils while *Acidobacteria* increase. These patterns are likely due to characteristics, such as cell envelope structure, that are shared by all members of a phylum and which convey selective fitness advantages in certain habitats. However, units of diversity defined by SSU rRNA gene sequences are insensitive to diversification resulting from dispersal limitation (5).

The examination of dispersal limitation is facilitated by analyzing discrete lineages at sufficient genetic resolution to resolve recent evolutionary divergence due to genetic drift (5, 12). Power to detect dispersal limitation is further increased by examining taxa from a single type of habitat that can be found distributed across large spatial scales (5). This level of focus is readily achieved through analysis of microbial strains that can be cultivated in isolation (13). For example, multilocus sequence analysis (MLSA) of *Sulfolobus* species indicates dispersal limitation and allopatric diversification on local and regional scales (14–16). In addition, genomic analysis of the soilborne pathogen *Burkholderia* *pseudomallei* revealed genetic diversification driven by vicariance along Wallace’s line between Southeast Asia and Australia (17). Also, analysis of single nucleotide polymorphisms in *Bacillus anthracis* indicated introduction of this soilborne pathogen to North America as a consequence of animal migrations during the late Pleistocene (18).

We chose the genus *Streptomyces* (phylum *Actinobacteria*) as a model system for exploring bacterial biogeography in soil. There are currently 615 described species of Streptomyces belonging to 130 clades (19). *Streptomyces* organisms are ubiquitous in soils, where they play an important role in the carbon cycle, particularly in the degradation of insoluble polymers, such as cellulose and chitin (20). These bacteria are also a major source for the discovery of clinically useful antibiotics and secondary metabolites (21). They produce spores that are resistant to starvation, UV light, and desiccation (22), and so they have the potential for widespread dispersal. Furthermore, analysis of the genetic diversity of individual species revealed that *Streptomyces* species can be distributed across large geographic regions (on scales of hundreds to thousands of kilometers [23, 24]).

Despite the prevalence and importance of Streptomyces species in soil, studies of their biogeography and evolutionary history are limited. Analysis of *Streptomyces* diversity on local spatial scales suggests that their diversity is influenced by environmental gradients (25, 26). Furthermore, analysis of *Streptomyces* diversity with respect to antibiotic production and resistance indicates that these phenotypes exhibit regional endemism, suggesting dispersal limitation and regional adaptation (27). We characterized Streptomyces strains from soils across the United States to determine whether their diversity scales with geographic distance and to examine the ecological and evolutionary factors that govern their biogeography. Our focus on a widespread spore-forming organism provides a strong test for the hypothesis of panmixia.

## RESULTS AND DISCUSSION

A total of 924 *Streptomyces* strains were isolated and characterized from 15 sites spanning the United States (see Table S1 in the supplemental material). Sites were selected to represent a narrow range of ecological characteristics, including meadow, pasture, or native grasslands dominated by perennial grasses and having moderately acidic soil (pH 6.0 ± 1.0 [average ± standard deviation, or SD]). Strains were isolated under uniform conditions (see Materials and Methods), which were used to select for strains having similar physiological characteristics. The analysis of physiologically similar strains from ecologically similar sites improves our ability to detect biogeographical patterns that result from drift by minimizing the importance of selection (reviewed in reference 5).

The isolated strains encompassed 208 unique rpoB sequences, which were classified into 107 OTUs with clusters defined at a patristic distance of 0.01 (OTU_rpoB_) (see Fig. S1 in the supplemental material). This distance has previously been observed to roughly correlate with species boundaries for *Streptomyces* (28). Good’s coverage was 0.88 for unique rpoB sequences and 0.95 for OTU_rpoB_, indicating high coverage of *Streptomyces* taxonomic diversity as captured under our isolation conditions (see Fig. S2 in the supplemental material). An average of 15.5 ± 8.5 OTU_rpoB_ was observed in each site. None of the OTU_rpoB_ had cosmopolitan distribution, and each OTU_rpoB_ occurred in an average of 1.7 ± 1.3 sites, with the most widespread taxon found in 8 sites. Identical *rpoB* sequences were observed in sites separated by more than 5,000 km (sites MS and AK2), indicating the potential for long-range dispersal.

**FIG 1:**
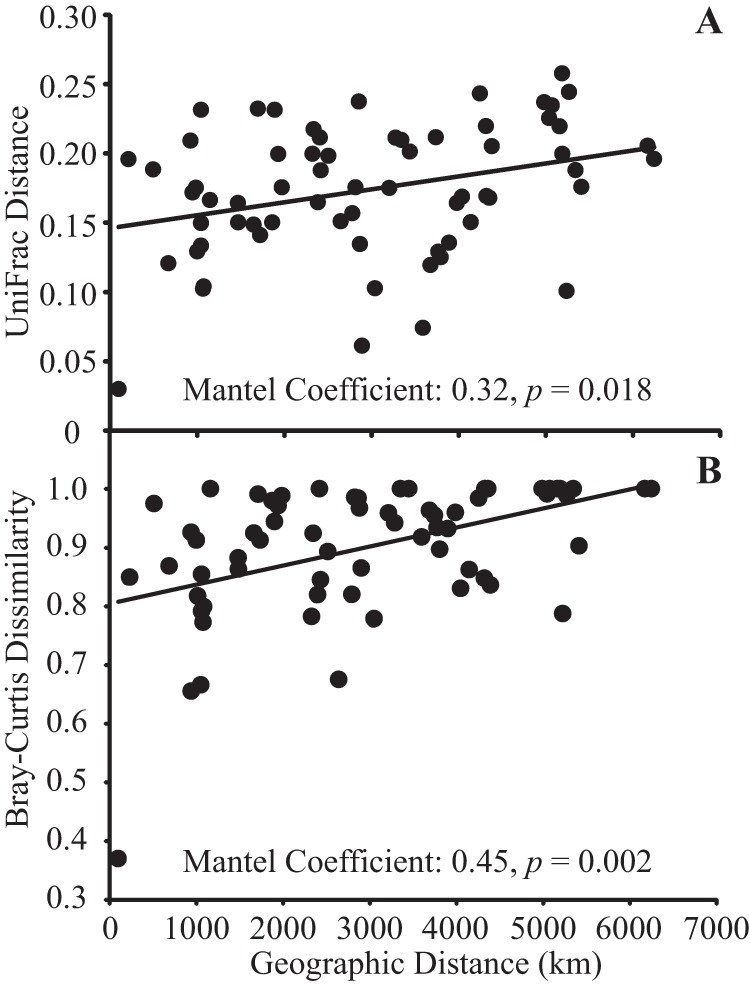
Phylogenetic (A) and taxonomic (B) dissimilarities of *Streptomyces* increase as a function of geographic distance between sites. The Mantel coefficient is provided along with the linear regression line.

Despite the potential for long-range dispersal, *Streptomyces* beta diversity varied in relation to geographic distance on spatial scales of 1,000 to 7,000 km (Fig. 1). The null model of random taxon assortment between sites was rejected, indicating that taxon composition differed significantly between sites (UniFrac analysis; *P* < 0.01). Both phylogenetic differentiation and Bray-Curtis dissimilarity (BCD) increased in relation to geographic distance (Fig. 1) (Unifrac distance, Mantel *R*^2^ = 0.32, *P* = 0.018; BCD for OTU_rpoB_, Mantel *R*^2^ = 0.45, *P* = 0.002; BCD for unique *rpoB*, Mantel *R*^2^ = 0.51, *P* = 0.002); these relationships remained significant if the Alaska (AK) sites were excluded from the analysis. In addition, we observed significant phylogenetic clustering in all sites except for the California, Mississippi, Texas, North Carolina, and Wisconsin sites (see Table S1 in the supplemental material). Phylogenetic clustering indicated that taxa within regions are more closely related to each other than they are to taxa from distant regions (29). These results agreed with our previous observations that *Streptomyces* populations can have large geographic ranges (23, 24) and with other observations that *Streptomyces* and other soil microorganisms exhibit dispersal limitation and regional endemism (27, 30–32).

While dispersal limitation is a parsimonious explanation for these results, an alternative hypothesis is panmixia and habitat filtering. This is better known to microbiologists as the Baas Becking hypothesis: “Everything is everywhere, but, the environment selects” (3). Habitat filtering of panmictic species would cause beta diversity to correspond strongly with environmental variables at spatial scales that fall within the Darwinian-Hutchinsonian zone (33), in which dispersal occurs more rapidly than diversification. If environmental characteristics are correlated with geographic distance within the Darwinian-Hutchinsonian zone, then habitat filtering could produce strong relationships between beta diversity and geographic distance. However, our sampling design reduced habitat variation and physiological variation of strains, and this should have likewise reduced the potential for habitat filtering to explain our results. In addition, observation of phylogenetic clustering across scales of thousands of kilometers is indicative of biogeographic rather than ecological processes, as dispersal limitation and regional diversification cause the species within regions to be more related to each other than they are to species from other regions (29, 33). Talbot et al., who likewise sampled soils associated with a single vegetation type (*Pinaceae*), also found little impact of environmental variation on large spatial scales when they examined fungal biogeography across North America (32).

To determine whether environmental characteristics could explain the patterns of beta diversity we observed, environmental variables were used to calculate the environmental distance between sites. Bray-Curtis distance varied in relation to the environmental distance between sites (Mantel *R*^2^ = 0.275, *P* = 0.008), but this result was not significant if the Alaska sites were excluded (Mantel *R*^2^ = 0.157, *P* = 0.146). Canonical correspondence analysis found no significant correlation between beta diversity and environmental characteristics whether the Alaska sites were included (*P* = 0.73) or excluded (*P* = 0.14). With respect to discrete environmental variables, *Streptomyces* beta diversity varied in relation to latitude, temperature, and soil pH, but not in relation to soil organic matter or annual precipitation (Table 1). Support for a relationship between beta diversity and temperature declined when the Alaska sites were excluded, though other results were largely unaffected by removing the Alaska sites (see Table S2 in the supplemental material). These results indicated that environmental variables have a minor though significant impact on *Streptomyces* beta diversity, with the greatest amount of variation due to latitude and soil pH. Soil pH is well known to impact the beta diversity of *Actinobateria* in soil (34, 35), and certain *Streptomyces* species are known to have habitat constraints which are circumscribed by soil pH (36).

**TABLE 1:** Relationships between environmental factors and *Streptomyces* phylogenetic (UniFrac distance) and taxonomic (Bray-Curtis dissimilarity) values^a^

Analysis type	Sequence inclusion	Correlation (*R*^2^ value) with^b^:				
		Latitude	Soil pH	Temp	SOM	PPT
UniFrac	Weighted	**0**.52**	0.35	**0**.50**	0.26	0.28
	Unweighted	**0**.48***	**0**.41*	**0**.44**	0.32	0.34
Bray-Curtis	Weighted	**0**.46***	**0**.40*	**0**.44**	0.21	0.33
	Unweighted	**0**.46***	**0**.44**	**0**.44**	0.25	0.31

a Analyzed by using the adonis program (permutational multivariate analysis of variance) within the R package.

b Bold values indicate statistically significant correlations (*, *P* < 0.05; **, *P* < 0.01; ***, *P* < 0.001). The analyses were performed by either including all *rpoB* sequences (weighted) or excluding duplicate sequences for each OTU (unweighted). Abbreviations: Temp, average annual temperature; SOM, soil organic matter content; PPT, annual average precipitation.

**FIG 2:**
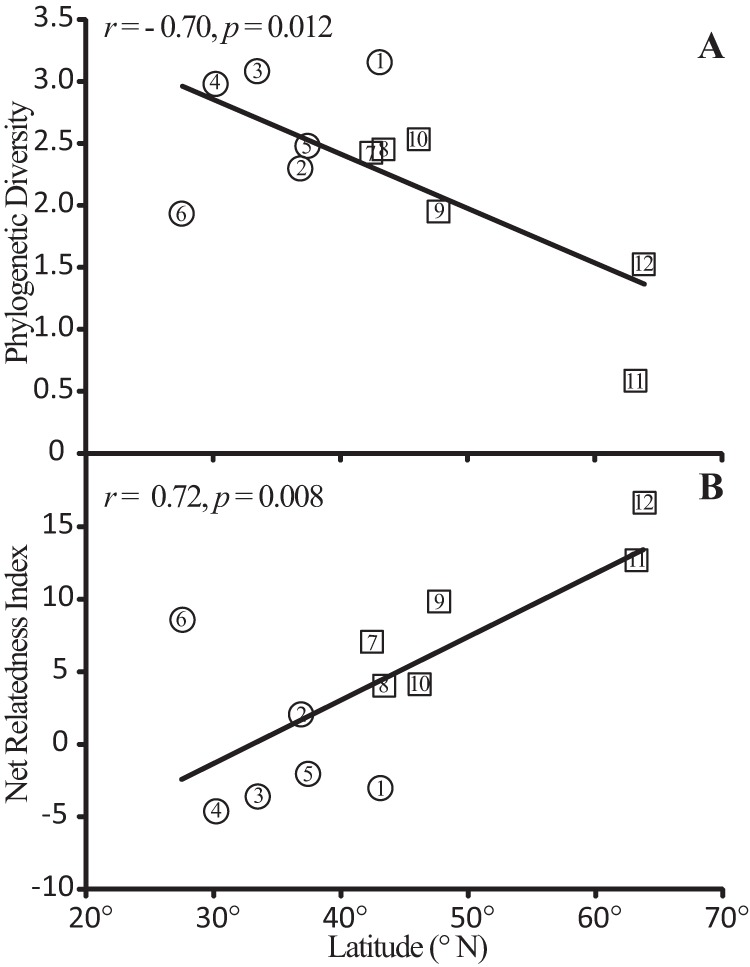
Latitude correlates with the phylogenetic diversity of *Streptomyces* as measured by both Faith’s phylogenetic diversity (A) and the net relatedness index (B). The Pearson correlation coefficient is provided along with the linear regression line. Symbols indicate the presence (□) or absence (○) of glaciation during the late Pleistocene. Numbers rank sites by time available for colonization, as follows: 1, WI; 2, NC; 3, MS; 4, TX; 5, CA; 6, FL; 7, NY; 8, ME; 9, WA; 10, OR; 11, AK1; 12, AK2. The FL site was below sea level at the beginning of the late Pleistocene, and sites in southern Wisconsin bound the Driftless Area, which escaped glaciation and has remained above sea level since the late Paleozoic.

*Streptomyces* phylogenetic diversity was negatively correlated with latitude (Table 2; Fig. 2). A latitudinal gradient was observed in relation to Faith’s phylogenetic diversity (PD) (Fig. 2A) (*r* = −0.70, *P* = 0.012), the net relatedness index (NRI) (Fig. 2B) (*r* = 0.72, *P* = 0.008), and mean root distance (MRD) (*r* = 0.61, *P* = 0.035). The correlation between *Streptomyces* phylogenetic diversity and latitude corresponded with the slope of the latitudinal diversity gradient observed for a wide range of taxa (average slope, −0.73), as determined in the meta-analysis reported by Hillebrand (37). As expected, *Streptomyces* diversity was also observed to correlate with temperature (which correlates strongly with latitude), but a significant correlation was not observed between phylogenetic diversity (as defined by PD, NRI, or MRD) and soil pH, or between soil pH and latitude (Table 2). Hence, while soil pH does explain some variation in beta diversity, it does not underlie the latitudinal diversity gradient we observed.

**TABLE 2:** Correlation coefficients for relationships between *Streptomyces* diversity and environmental characteristics across sites^a^

Characteristic	Correlation (*R*^2^ value) based on:							
	PD	NRI	MRD	Lat.	Long.	Temp	SOM	PPT
NRI	**−0**.86**							
MRD	**−0**.70*	**0**.67*						
Lat.	**−0**.70*	**0**.72**	**0**.61*					
Long.	**0**.62*	−0.51	−0.54	−0.75**				
Temp	**0**.61*	**−0**.64*	−0.47	−0.97**	**0**.60*			
SOM	0.45	−0.24	−0.14	−0.16	−0.06	0.27		
PPT	0.42	−0.24	0.01	−0.40	0.54	0.38	0.25	
pH	0.16	−0.39	−0.42	−0.45	0.11	0.43	0.09	−0.41

a Bold values indicate statistically significant correlations (*, *P* < 0.05; **, *P* < 0.01). Abbreviations: Lat., latitude; Long., longitude; Temp, average annual temperature; SOM, soil organic matter content; PPT, annual average precipitation; pH, soil pH.

The latitude diversity gradient is a fundamental pattern in ecology, has been well-documented since the days of Wallace (38), and is consistent across a wide taxonomic range of plant and animal species (37). Far fewer studies have been able to document a latitude diversity gradient in bacteria. The most likely explanation for this absence of evidence is the low taxonomic resolution at which microbial diversity is typically measured (5). Latitudinal patterns of diversity are typically observed at the population to class level in plants and animals (38). And yet, while the domain *Bacteria* is far older and far more diverse than the domain *Eukarya*, it is common practice to quantify patterns of microbial diversity for all *Bacteria* as if this domain represented a single ecologically coherent unit. However, the ecological and evolutionary factors that cause patterns of diversity are best understood when we quantify diversity at the level of taxonomic resolution that is sufficient to describe processes of diversification (5).

Taxon-specific, culture-dependent approaches to describing microbial diversity allow us to evaluate the processes that contribute to microbial diversification. The notable limitations of cultivation-dependent approaches are the potential for bias against certain physiological traits and the lack of sensitivity to taxa present at very low relative abundance. For example, the cultivation method we used would be unable to detect strains present at less than 1.25 × 10^5^ cells per g of soil. The biogeographic sampling of plants and animals is likewise subject to both veil line effects that obscure the presence of rare taxa and to sampling artifacts that may bias samples against certain physiological types (for example, nocturnal species are rarely collected during the day). However, a distinct advantage of a taxon-specific approach is that it allows for the physiological and genotypic characterization of discrete microbial taxa. Such data are highly valuable, because hypotheses that describe patterns of diversification often make predictions that can be tested with physiological and genomic data. Hence, taxon-specific, culture-dependent approaches provide evidence suitable for evaluating hypotheses derived from the broader field of evolutionary ecology.

The evolutionary forces that generate latitudinal diversity gradients remain under debate, and several hypotheses have been proposed to explain this biogeographical pattern (reviewed in references 39 and 40). Ecological hypotheses explain differences in species richness as a function of ecological factors, such as carrying capacity, productivity, and niche availability, which vary across climate gradients (41). Evolutionary tempo hypotheses invoke the relationship between higher temperatures and increased kinetics of metabolism to predict that evolutionary rates and cladogenesis vary across temperature gradients (42). Hypotheses based on historical contingency propose that diversity gradients result from historical geologic, ecological, or demographic events that influenced dispersal and diversification (40, 43, 44).

The niche conservatism hypothesis explains the latitude diversity gradient as a function of historical climate change (45–47). Specifically, the hypothesis posits that climate oscillations and patterns of glaciation have constrained time for speciation at higher latitudes and that species found at higher latitudes are derived as a result of demographic range expansion from species occupying lower latitudes. Niche conservatism is facilitated by high-density blocking, whereby early colonists subsequently impose density-dependent barriers to colonization by late dispersing individuals (48). Furthermore, derived populations at higher latitudes are expected to evolve adaptations to their new habitats, thereby imposing further barriers to latitudinal dispersal across climate regimes (48). Glacial retreat at the end of the Pleistocene caused demographic range expansions in diverse plant and animal species, and the legacy of these events is readily observed in extant patterns of genetic diversity (49–53). In addition, dispersal limitation and strong priority effects suggest that historical contingency has played an important role in determining the diversity of soil fungal communities (54, 55), and this may explain why the diversity of soil fungi varies with respect to the biogeographic provinces described for North America (32).

The Wisconsin Glacier occurred between 30,000 and 10,000 years BP (maximum extent, approximately 15,000 to 16,000 years BP) and covered nearly all of Canada and the northern part of the United States except for a small region in southwestern Wisconsin known as the Driftless Area (56, 57). The legacy of Pleistocene glaciation in North America can often be observed as discontinuity in genetic diversity, which manifests at roughly 40°N latitude (58). In several instances, the genetic diversity of terrestrial species of *Bacteria* has been shaped by glacial dynamics during the late Pleistocene (18, 59), including evidence for discontinuity in the genetic diversity of *Actinobacteria* along a chronosequence formed by recession of Wisconsin glaciation (60).

The latitudinal gradient of diversity we observed for *Streptomyces* is broadly consistent with the niche conservatism hypothesis. The niche conservatism hypothesis predicts that latitude will correlate negatively with phylogenetic diversity and positively with phylogenetic clustering and mean root distance (61, 62), and these predictions are consistent with our observations for the *Streptomyces* diversity gradient (Table 2; Fig. 2). As predicted by the niche conservatism hypothesis, PD is lower (glaciated, 1.9 ± 0.8; nonglaciated, 2.7 ± 0.5; *P* = 0.071), and both NRI phylogenetic clustering (glaciated, 10.0 ± 4.9; nonglaciated, −0.5 ± 5.0; *P* = 0.008) and MRD (glaciated, 26.1 ± 3.0; nonglaciated, 22.1 ± 2.7; *P* = 0.037) are higher in sites subjected to glaciation than in nonglaciated sites. In addition, we observed a correlation between diversity and time available for colonization with respect to phylogenetic diversity (Spearman’s *r* = 0.64, *P* = 0.026), net relatedness index (Spearman’s *r* = −0.82, *P* = 0.001), and mean root distance (Spearman’s *r* = 0.70, *P* = 0.012), and this is also a prediction of niche conservatism (see Table S1 in the supplemental material).

**FIG 3:**
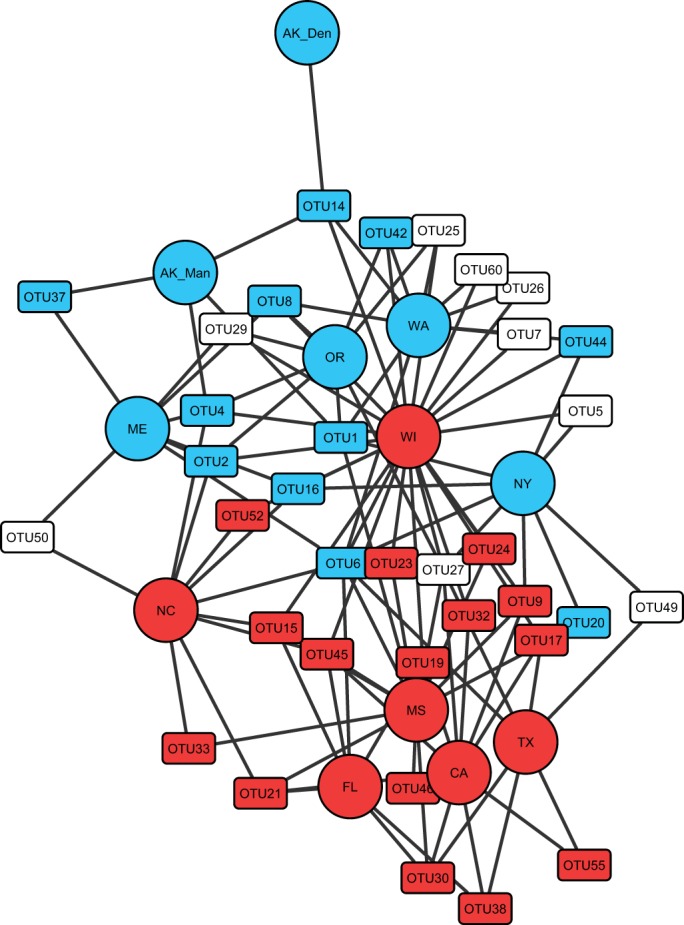
Network analysis illustrating OTU_rpoB_ sharing across sample sites, indicating a latitudinal gradient of beta diversity consistent with glaciation history. Sample sites (circles) are colored by history of glaciation (blue) or nonglaciation (red) during the late Pleistocene. OTUs (rectangles) are colored if both >90% of sequences were recovered from glacial (blue) or nonglacial (red) sites and the null hypothesis of random assortment was rejected (Fisher exact test, *P* < 0.05). The WI site was the most highly connected site in the network and has previously been proposed as a glacial refugium for certain plant species, as discussed in the text. OTUs found in only one site were excluded from the analysis.

The niche conservatism hypothesis predicts that sites at higher latitudes were colonized following glacial retreat as a result of demographic range expansion from species at lower latitudes. Network analysis of OTU_rpoB_ sharing between sites indicated a strong latitudinal delineation in the OTU_rpoB_ compositions of sites (Fig. 3). The network showed that 95% (21/22) of the shared OTUs observed in glaciated sites were also found in a nonglaciated site, and most of these OTUs (18/22) were common to Wisconsin. The WI site also stood out in relationships between NRI, PD, and latitude (Fig. 2), having lower NRI and PD than sites of comparable latitude (NY and ME sites) and being the only northern latitude site to lack significant phylogenetic clustering (see Table S1 in the supplemental material). We note that the Driftless Area in Wisconsin has been proposed as a refugium for certain species of plants (63), and the unusual diversity of *Streptomyces* in this region may be due to the fact that much of Wisconsin was never glaciated and thus strains from this region had greater opportunity than lower-latitude strains to disperse into habitat exposed by glacial retreat. In addition, niche conservatism posits that time for speciation limits diversity, and hence it is interesting that the FL site was below sea level during the last interglacial period, as recently as 118,000 years ago (64). The FL site, despite being at the lowest latitude, also had the lowest phylogenetic diversity of any nonglacial site and was the only nonglacial site to demonstrate significant phylogenetic clustering (Fig. 2; see also Table S1).

Finally, network analyses of haplotype phylogeography provided evidence for the regional diversification of *Streptomyces* clades as a consequence of population expansion and isolation by distance (see Fig. S3 and Table S3 in the supplemental material). The largest observed rpoB haplotype network represented 156 strains. Nested clade analysis revealed that the pattern of ancestry in this network was consistent with divergence due to population expansion (representing 82% of the strains in the network) and subsequent isolation of clades by distance (representing 95% of the strains in the prior subset) (see Fig. S3 and Table S3). Overall, 421 strains belonged to clades whose pattern of ancestry was consistent with dispersal limitation, and these represented 91% of the strains in networks that supported evolutionary inference (see Fig. S3 and Table S3). These findings are consistent with the hypothesis that historical demographic processes explain patterns of *Streptomyces* biogeography.

Strong correlations between latitude, temperature, and climate make it difficult to determine the ultimate mechanisms that generate latitudinal diversity gradients. For example, the kinetic effects of temperature have been previously shown to impact latitudinal diversity gradients for marine bacteria (65). In addition, the diversity gradient we observed could be due to an ecological correlation between *Streptomyces* diversity and unmeasured differences in biotic or abiotic variables at our sites. However, the ability of temperature to increase evolutionary tempo would not explain the low diversity in our FL site or the high diversity in Wisconsin. Likewise, neither the effects of temperature nor the effects of biotic or abiotic variables would readily predict discontinuity in phylogenetic clustering or mean root depth with respect to historically glaciated and nonglaciated sites; nor would these hypotheses explain well the inference of demographic expansion provided by analysis of haplotype networks. Finally, observations of the genetic diversity within *Streptomyces* populations indicate that, despite evidence for dispersal limitation, multiple species are in linkage equilibrium across large latitudinal gradients (23, 24). The most parsimonious explanation of these apparently conflicting results is that these populations have experienced an evolutionarily recent demographic range expansion from low to high latitudes. While the results we describe are broadly consistent with niche conservatism, they do not rigorously exclude other hypotheses. Ultimately, it is likely that latitudinal diversity gradients can result from a combination of ecological, evolutionary, and historical processes and that the relative importance of these different mechanisms varies between different taxa.

In summary, these data indicate that *Streptomyces* diversity varies in relation to geographic distance and manifests in a latitudinal diversity gradient. Furthermore, the data suggest that these patterns result from dispersal limitation and the regional diversification of clades. Habitat filtering is often invoked to explain microbial biogeography, but while habitat filtering could produce patterns of beta diversity that vary with latitude, this hypothesis does not predict latitudinal gradients of PD, NRI, and MRD. While soil pH was shown to influence beta diversity (Table 1), soil pH did not correlate with latitude across our sites, and soil pH did not correlate with patterns of PD, NRI, and MRD (Table 2). Hence, we conclude that soil pH is unlikely to underlie the latitudinal diversity gradient. The hypothesis that is most parsimonious given the data we have described is that historical demographic events underlie the *Streptomyces* latitudinal diversity gradient, though these data do not exclude completely either evolutionary or ecological hypotheses. The hypothesis of niche conservatism leads to specific predictions about the genetic consequences of demographic expansion and the phylogenetic conservation of phenotypic traits associated with different climate regimes (45–47). Physiological and genomic analyses of *Streptomyces* strains from our culture collection should make it possible to further test specific predictions of niche conservatism.

## MATERIALS AND METHODS

### Sampling and strain collection.

*Streptomyces* strains (*n* = 924) were isolated from 15 sites across the United States (see Table S1 in the supplemental material). Soils were collected exclusively from sites dominated by perennial grasses with neutral to acidic pH (lawn, meadow, and pasture). Soils were sampled at a 0- to 5-cm depth and air dried at room temperature. Soil organic matter content was measured by loss on ignition, and soil pH was determined for a 1:2 (wt/vol) dilution of soil in 0.01 M CaCl_2_ (66). Precipitation and temperature data were obtained from the U.S. National Centers for Environmental Information (http://www.ncdc.noaa.gov/) and represent 30-year climate normal data unless otherwise described. Glacial extent and chronology were determined based on the methods of Peltier (67).

For *Streptomyces* isolation, 50 mg of soil was diluted 1:100 (wt/vol) in phosphate-buffered saline and mixed vigorously (1 to 2 min), after which 25 to 50 µl was spread onto glycerol-arginine plates containing 300 mg/liter cycloheximide and 30 mg/liter Rose bengal (68, 69), with the pH adjusted to 8.7 as previously described (23). Colonies developed after 5 to 7 days of incubation at room temperature, and strains were isolated by repeated streaking. DNA was extracted from purified cultures, which were grown with shaking at 30°C in liquid yeast extract-malt extract medium (YEME) containing 0.5% glycine (22), using a standard phenol-chloroform-isoamyl alcohol protocol (70). The resulting DNA was resuspended in 150 µl of Tris-EDTA buffer.

### Sequence analysis.

PCR amplification and sequencing of *rpoB* was performed as described elsewhere (71). Briefly, PCR was performed in 25-µl volumes containing 1× AmpliTaq gold buffer (Applied Biosystems, Foster City, CA), 3 mM MgCl_2_, 2.5 mM each deoxynucleoside triphosphate (Promega, Madison, WI), 0.4 µM *rpoB* forward primer (5′-GAGCGCATGACCACCCAGGACGTCGAGGC-3′), 0.4 µM *rpoB* reverse primer (5′-CCTCGTAGTTGTGACCCTCCCACGGCATGA-3′), 10% dimethyl sulfoxide, 1.25 U AmpliTaq Gold (Applied Biosystems, Foster City, CA), and 50 to 200 ng DNA. The following reaction conditions were used: 95°C for 10 min for initial denaturation, followed by 35 cycles of 95°C for 20 s, 65°C for 30 s, and 72°C for 45 s, and final extension at 72°C for 10 min. Sequencing of PCR products was performed at the Cornell Life Sciences Core Laboratories Center. Sequences were assembled manually, and trace files were inspected visually and uniformly trimmed to achieve a final length of 377 bp.

Sequences used in phylogenetic analyses were aligned using MUSCLE (72). Maximum likelihood trees were constructed with the general time-reversible model of nucleotide substitution (73), incorporating an estimated proportion of invariant sites and discrete gamma distribution (GTR+I+G) within the RAxML program (74). Trees were rooted using *Mycobacterium smegmatis* as the outgroup.

### Analyses of *Streptomyces* diversity and phylogeography.

Operational taxonomic units based on *rpoB* sequences were defined at a 0.01 nucleotide dissimilarity cutoff using patristic distances as implemented in RAMI (75). This dissimilarity cutoff roughly delineates the genetic divergence between characterized *Streptomyces* species (28). In the case of the Wisconsin and North Carolina sites, sequences were aggregated across two or three soil samples, respectively, to ensure that >30 strains were available to represent each region. The decision to aggregate was justified by similarities in climate, geography, and soil characteristics across the aggregated samples. In addition, previous analyses had indicated that *Streptomyces* species are broadly distributed at regional scales (>1,000 km) (23, 24). This provided 12 sites with 77 ± 30 (mean ± SD) strains characterized per site. Beta diversity was evaluated through hierarchical clustering implemented within UniFrac (76). Mantel correlations between matrices of geographic distance and either UniFrac or Bray-Curtis distances were performed with the R package ecodist (77) and the Pearson correlation method with 1,000 permutations. Patterns of OTU_rpoB_ sharing were visualized using Cytoscape 2.8 and the y-files organic layout (78).

Values for the NRI, nearest taxon index (NTI), and Faiths PD were calculated using Phylocom v.4.2 (79). For both the NRI and NTI, positive values indicate phylogenetic clustering (i.e., closely related taxa cooccur more than expected by chance), negative values indicate overdispersal or phylogenetic evenness, and values close to zero suggest a phylogenetically random assembly of species. Significance was determined by permutation (*n* = 999) in comparison to a null model where taxa are assigned to each site by random draw without replacement from the list of all taxa. The MRD was calculated as the average number of nodes separating the species in a site from the root of their phylogenetic tree (80).

Haplotype networks were created using a statistical parsimony procedure (81, 82) as implemented in TCS v1.18 (83). Closed loops representing network ambiguities were resolved using the nesting rules proposed by Templeton et al. (82). The final nested clade information was used as input in the program GeoDis v2.2 (84). GeoDis analyzes the nested haplotype network to make inferences on the processes that could have produced the association of the haplotype distribution and geography. Both TCS v1.18 and GeoDis v2.2 were performed with the ANeCA platform (85).

### Nucleotide sequence accession numbers.

*rpoB* gene sequences determined in this study are available from GenBank under accession numbers KU238378:KU238472 and KU956103:KU956931.

## SUPPLEMENTAL MATERIAL

Table S1  *Streptomyces* strains isolated and characterized from the series of 15 sites described in the text (In the cases of Wisconsin and North Carolina, samples were aggregated across two or three soil samples, respectively, to represent each region. “Time,” the time available for colonization; mya, millions of years ago; SOM, soil organic matter. Asterisks identify NRI values that showed clustering [positive] or overdispersion [negative] that are unlikely to result from chance [*P* < 0.01].) Table S1, PDF file, 0.1 MB

Table S2  Results from Table 2 alongside results obtained when sites from Alaska were excluded (The column reporting “sites” indicates results obtained when Alaska sites were included [“+AK” rows are the same as those depicted in Table 2] or excluded [−AK]. Results that reveal different outcomes between the +AK and –AK analyses are indicated with shading. Relationships between environmental factors and *Streptomyces* phylogenetic [UniFrac distance] and taxonomic [Bray-Curtis dissimilarity] differences were analyzed by using adonis [permutational multivariate analysis of variance]. Values indicate *R*^2^ values, and results that are unlikely due to chance are indicated with asterisks [*, *P* < 0.05; **, *P* < 0.01; ***, *P* < 0.001]. The analyses were performed by either including all *rpoB* sequences [weighted] or excluding duplicate sequences for each OTU [unweighted].) Table S2, PDF file, 0.04 MB

Table S3  Significant inferences from nested clade analysis of *Streptomyces rpoB* haplotypes (see Fig. S3). Table S3, PDF file, 0.1 MB

Figure S1  Maximum likelihood tree based on rpoB sequences of *Streptomyces* strains. A total of 924 *Streptomyces* strains were isolated from 15 sites in North America and classified into 107 OTUs on the basis of *rpoB* sequence dissimilarity. The inner colored ring includes sequential color blocks to indicate the 107 OTU_rpob_ identified in this study. The outer colored ring identifies the history of the site from which each strain was isolated, as indicated in the color scale (FL and WI are indicated as well, due to their distinctive geologic history). The site from which each strain was isolated can be determined by the strain name, as described in Table S1. Download Figure S1, PDF file, 0.9 MB

Figure S2  The rarefied collectors curve indicates that OTU_rpoB_ were well sampled from the sampling sites (with respect to the constraints imposed by strain collection). Good’s coverage was 0.88 for unique *rpoB* sequences and 0.95 for OTU_rpoB_. Download Figure S2, PDF file, 0.3 MB

Figure S3  Nested clade analysis of Streptomyces rpoB haplotype networks provides evidence for contiguous range expansion and dispersal limitation. Circles represent rpoB haplotypes, with radii proportional to the number of strains that belong to the haplotype. Haplotypes are shaded to represent strain source, with the fraction of strains isolated from previously glaciated or nonglaciated sites indicated in blue and red, respectively. Each line symbolizes one mutational step, with dots indicating inferred haplotypes not sampled. The complete set of haplotype networks is shown (Table S2 shows the evolutionary inferences for each clade). Download Figure S3, PDF file, 1 MB
