# Supplementary material for: A Latitudinal Diversity Gradient in Terrestrial Bacteria of the Genus Streptomyces
Source: mBio. 2016 Apr 5;7(2):e02200-15. doi: 10.1128/mBio.02200-15 (PMC4817263; doi:10.1128/mBio.02200-15)
Supplement: Table S1 — Streptomyces strains isolated and characterized from the series of 15 sites described in the text (In the cases of Wisconsin and North Carolina, samples were aggregated across two or three soil samples, respectively, to represent each region. “Time,” the time available for colonization; mya, millions of years ago; SOM, soil organic matter. Asterisks identify NRI values that showed clustering [positive] or overdispersion [negative] that are unlikely to result from chance [P < 0.01].) [file mbo002162750st1.pdf]

Table S1. *Streptomyces* strains were isolated and characterized from a series of 15 sites as described. In the case of WI and NC, samples were aggregated across two or three soil samples respectively to represent each region. Abbreviations are: phylogenetic diversity (PD), net relatedness index (NRI), time available for colonization (Time), millions of years ago (mya), soil organic matter (SOM), average (ave.). Asterisks are used to identify values of NRI that show clustering (positive) or over dispersion (negative) that are unlikely to result from a chance ($p < 0.01$).

Site	Sampling ID	Strain number	OTU obs.	Lat. (°N)	Long. (-°W)	PD	NRI	MRD	Time (mya)	Rank age	Soil pH	Ave. annual temp. (°C)	SOM (%)	Ave. annual precip. (cm)	Strain names
WI	Aggregate	87	20	43.11	88.69	3.15	-3.05*	20.3	500	1	6.65	7.85	7.70	90.3	
	Sun Prairie	65	n.d.	43.17	89.24	n.d.	n.d.	n.d.	n.d.	n.d.	6.59	8.37	6.13	92.5	sun
	Brookfield	22	n.d.	43.06	88.13	n.d.	n.d.	n.d.	n.d.	n.d.	6.79	7.33	9.26	88.0	b
NC	Aggregate	55	15	36.87	79.34	2.30	2.04	24.4	300	2	6.40	15.54	5.72	111.9	
	Charlotte	27	n.d.	38.81	78.26	n.d.	n.d.	n.d.	n.d.	n.d.	6.40	n.d.	n.d.	n.d.	ch
	Uwharrie	19	n.d.	35.71	79.88	n.d.	n.d.	n.d.	n.d.	n.d.	n.d.	15.54	5.72	120.2	uw
	Greensboro	9	n.d.	36.09	79.89	n.d.	n.d.	n.d.	n.d.	n.d.	n.d.	n.d.	n.d.	n.d.	gb
MS	Starkville	90	13	33.461	88.8	3.08	-3.63*	20.6	80	3	6.82	17.16	12.84	153.3	ms
TX	Austin	42	16	30.204	97.77	3.00	-4.64*	25.1	50	4	7.29	21.04	8.14	86.7	t
CA	Palo Alto	34	20	37.429	122.17	2.48	-2.08	18.3	3	5	7.11	14.46	6.26	37.2	st
FL	Fort Pierce	70	6	27.54	80.35	1.93	8.57*	23.8	0.118	6	7.09	22.87	5.64	124.7	f
NY	Ithaca	142	11	42.45	76.46	2.42	7.04*	21.2	0.018	7	5.20	8.17	3.49	99.8	# only
ME	Kennebunk	85	10	43.401	70.54	2.45	4.02*	24.5	0.018	8	4.76	7.14	5.74	139.8	me
WA	Bothell	105	9	47.731	122.24	1.95	9.82*	25.9	0.014	9	5.31	11.36	11.34	83.1	wa
OR	Astoria	79	13	46.18	123.85	2.53	4.12*	27.3	0.013	10	3.90	10.81	8.23	163.5	or
AK1	Denali	40	1	63.22	147.68	0.58	12.67*	30	0.013	11	6.00	-3.58	1.89	61.3	den
AK2	Manley Springs	95	5	63.87	149.02	1.52	16.64*	27.4	0.013	12	6.00	-3.92	9.96	58.2	man
