# Supplementary material for: A Latitudinal Diversity Gradient in Terrestrial Bacteria of the Genus Streptomyces
Source: mBio. 2016 Apr 5;7(2):e02200-15. doi: 10.1128/mBio.02200-15 (PMC4817263; doi:10.1128/mBio.02200-15)
Supplement: Table S2 — Results from Table 2 alongside results obtained when sites from Alaska were excluded (The column reporting “sites” indicates results obtained when Alaska sites were included [“+AK” rows are the same as those depicted in Table 2] or excluded [−AK]. Results that reveal different outcomes between the +AK and –AK analyses are indicated with shading. Relationships between environmental factors and Streptomyces phylogenetic [UniFrac distance] and taxonomic [Bray-Curtis dissimilarity] differences were analyzed by using adonis [permutational multivariate analysis of variance]. Values indicate R2 values, and results that are unlikely due to chance are indicated with asterisks [*, P < 0.05; **, P < 0.01; ***, P < 0.001]. The analyses were performed by either including all rpoB sequences [weighted] or excluding duplicate sequences for each OTU [unweighted].) [file mbo002162750st2.pdf]

Table S2. Results from Table 2 are represented alongside results obtained when sites from Alaska are excluded. The column 'sites' indicates results obtained when Alaska sites are included (+AK, these rows are the same as those depicted in Table 2) or excluded (-AK). Results that provide different outcomes between the +AK and –AK analyses are indicated with shading. Relationships between environmental factors and *Streptomyces* phylogenetic (UniFrac distance) and taxonomic (Bray-Curtis dissimilarity) dissimilarity were analyzed by ADONIS (permutational multivariate analysis of variance). Values indicate R^2 and results that are unlikely due to chance are indicated with asterisks (*, $p < 0.05$; **, $p < 0.01$; ***, $p < 0.001$). The analyses were performed by either including all *rpoB* sequences (weighted) or excluding duplicate sequences for each OTU (un-weighted).

Analysis	Sequence Weighting	Sites	Latitude	Soil pH	Average temp.	SOM	Annual precip.
UniFrac	weighted	+AK	0.52**	0.35	0.50**	0.26	0.28
		-AK	0.38	0.43	0.35	0.33	0.25
	un-weighted	+AK	0.48***	0.41*	0.44**	0.32	0.34
		-AK	0.48*	0.51**	0.38	0.32	0.35
Bray-Curtis	weighted	+AK	0.46***	0.40*	0.44**	0.21	0.33
		-AK	0.44***	0.44**	0.41*	0.29	0.32
	un-weighted	+AK	0.46***	0.44**	0.44**	0.25	0.31
		-AK	0.48**	0.51**	0.42	0.24	0.33
