# Supplementary material for: A Latitudinal Diversity Gradient in Terrestrial Bacteria of the Genus Streptomyces
Source: mBio. 2016 Apr 5;7(2):e02200-15. doi: 10.1128/mBio.02200-15 (PMC4817263; doi:10.1128/mBio.02200-15)
Supplement: Table S3 — Significant inferences from nested clade analysis of Streptomyces rpoB haplotypes (see Fig. S3). [file mbo002162750st3.pdf]

Table S2. Significant inferences from nested clade analysis (Figure S3) of *Streptomyces rpoB* haplotypes.

Clade	Inference
1-2	Contiguous range expansion
1-20	Contiguous range expansion
1-72	Contiguous range expansion
1-73	Contiguous range expansion
1-75	Restricted dispersal by distance
2-5	Restricted dispersal by distance
2-65	Restricted dispersal by distance
2-70	Allopatric fragmentation
2-78	Allopatric fragmentation
2-100	Allopatric fragmentation
2-119	Allopatric fragmentation
2-121	Allopatric fragmentation
3-69	Restricted dispersal by distance
3-74	Insufficient resolution to distinguish between range expansion and restricted dispersal
4-1	Restricted dispersal by distance
4-2	Restricted dispersal by distance
4-3	Restricted dispersal by distance
4-71	Contiguous range expansion
4-74	Insufficient resolution to distinguish between range expansion and restricted dispersal
4-75	Insufficient resolution to distinguish between range expansion and restricted dispersal
5-1	Contiguous range expansion
5-2	Contiguous range expansion
