# Supplementary material for: A Latitudinal Diversity Gradient in Terrestrial Bacteria of the Genus Streptomyces
Source: mBio. 2016 Apr 5;7(2):e02200-15. doi: 10.1128/mBio.02200-15 (PMC4817263; doi:10.1128/mBio.02200-15)
Supplement: Figure S1 — Maximum likelihood tree based on rpoB sequences of Streptomyces strains. A total of 924 Streptomyces strains were isolated from 15 sites in North America and classified into 107 OTUs on the basis of rpoB sequence dissimilarity. The inner colored ring includes sequential color blocks to indicate the 107 OTUrpob identified in this study. The outer colored ring identifies the history of the site from which each strain was isolated, as indicated in the color scale (FL and WI are indicated as well, due to their distinctive geologic history). The site from which each strain was isolated can be determined by the strain name, as described in Table S1. Download [file mbo002162750sf1.pdf]

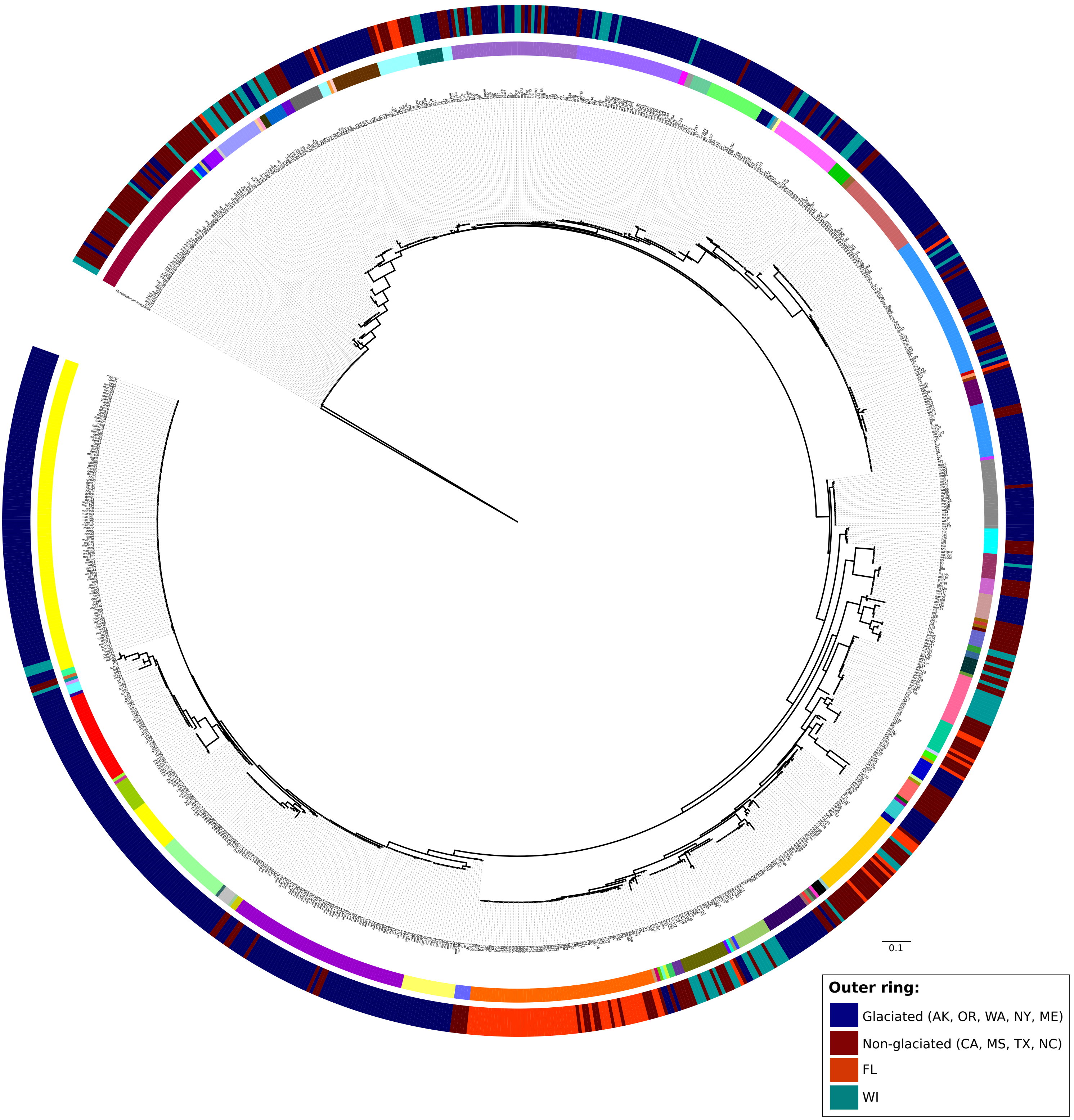

Figure S1. Maximum likelihood tree based on rpoB sequences of *Streptomyces* strains. A total of 924 *Streptomyces* strains were isolated from 15 sites in North America and classified into 107 OTU on the basis of *rpoB* sequence dissimilarity. The inner colored ring uses sequential color blocks to indicate the 107 OTU_{rpob} identified in this study. The outer color ring identifies the history of the site from which each strain was isolated as indicated in the color scale (FL and WI are indicated as well due to their distinctive geological history). The site from which each strain was isolated can be determined by the strain name as described in Table S1.
