# Supplementary material for: A Latitudinal Diversity Gradient in Terrestrial Bacteria of the Genus Streptomyces
Source: mBio. 2016 Apr 5;7(2):e02200-15. doi: 10.1128/mBio.02200-15 (PMC4817263; doi:10.1128/mBio.02200-15)
Supplement: Figure S2 — The rarefied collectors curve indicates that OTUrpoB were well sampled from the sampling sites (with respect to the constraints imposed by strain collection). Good’s coverage was 0.88 for unique rpoB sequences and 0.95 for OTUrpoB. Download [file mbo002162750sf2.pdf]

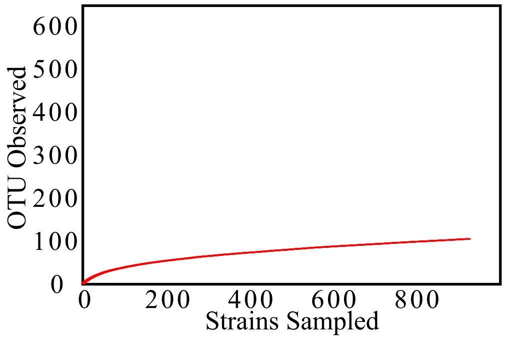

Figure S2. The rarefied collectors curve indicates that OTU_{rpoB} have been well sampled from the sampling sites (with respect to the constraints imposed by strain collection). Good's coverage is 0.88 for unique *rpoB* sequences and 0.95 for OTU_{rpoB}.
