# Supplementary material for: A Latitudinal Diversity Gradient in Terrestrial Bacteria of the Genus Streptomyces
Source: mBio. 2016 Apr 5;7(2):e02200-15. doi: 10.1128/mBio.02200-15 (PMC4817263; doi:10.1128/mBio.02200-15)
Supplement: Figure S3 — Nested clade analysis of Streptomyces rpoB haplotype networks provides evidence for contiguous range expansion and dispersal limitation. Circles represent rpoB haplotypes, with radii proportional to the number of strains that belong to the haplotype. Haplotypes are shaded to represent strain source, with the fraction of strains isolated from previously glaciated or nonglaciated sites indicated in blue and red, respectively. Each line symbolizes one mutational step, with dots indicating inferred haplotypes not sampled. The complete set of haplotype networks is shown (Table S2 shows the evolutionary inferences for each clade). Download [file mbo002162750sf3.pdf]

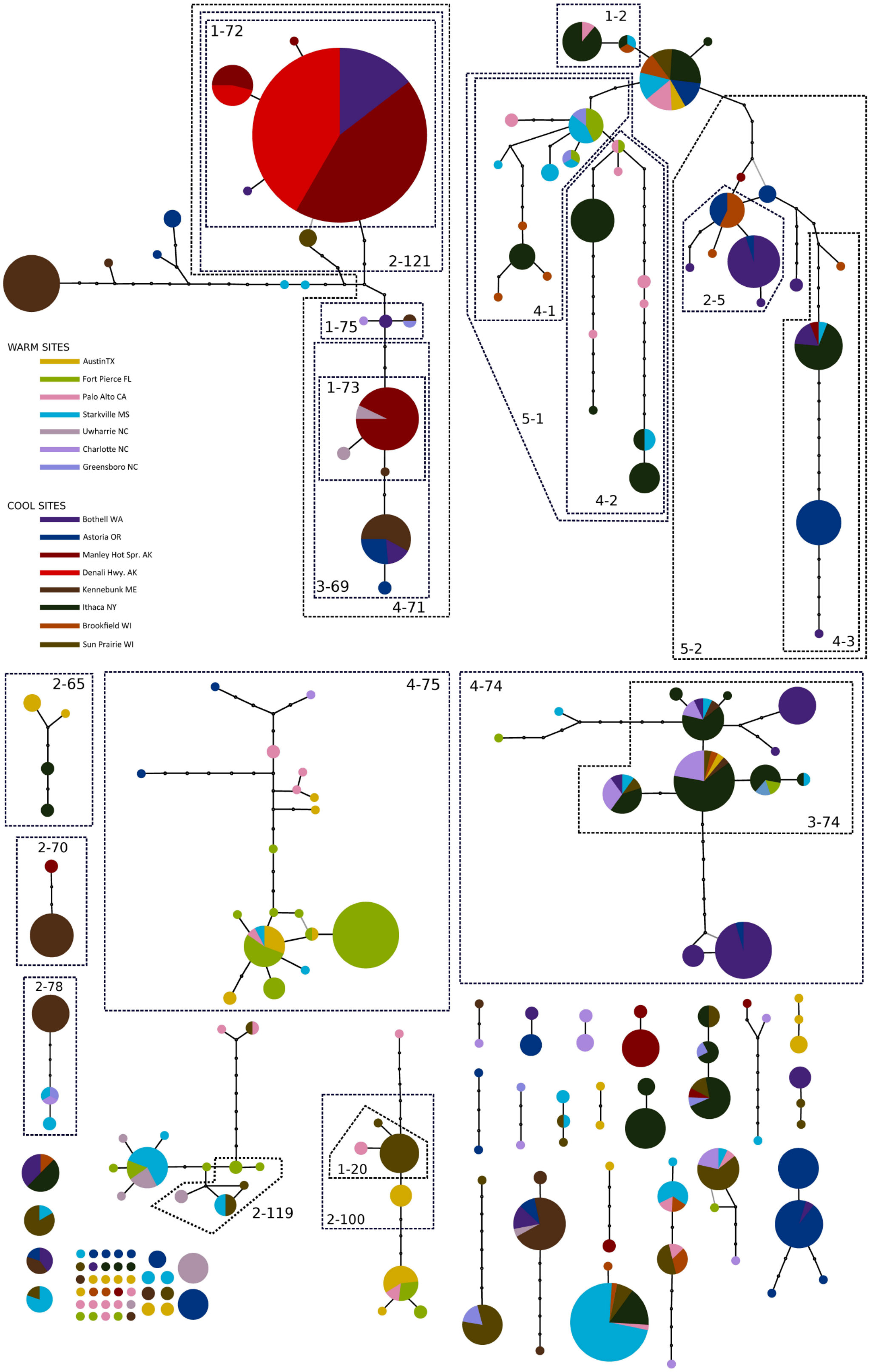

Figure S3. Nested clade analysis of *Streptomyces rpoB* haplotype networks provides evidence for contiguous range expansion and dispersal limitation. Circles represent *rpoB* haplotypes with radius proportional to the number of strains that belong to the haplotype. Haplotypes are shaded to represent strain source with the fraction of strains isolated from previously glaciated or non-glaciated sites indicated in blue and red respectively. Each line symbolizes one mutational step with dots indicating inferred haplotypes not sampled. Figure S3 shows the complete set of haplotype networks. Table S2 shows the evolutionary inferences for each clade.
